# Alive and on Initial Antifibrotic Treatment: Real-World Treatment Durability in Idiopathic and Progressive Pulmonary Fibrosis

**DOI:** 10.3390/jcm15218712

**Published:** 2026-11-08

**Authors:** Fatma Isil Uzel, Ferhan Karataş, Burak Uzel, Yüksel Peker

**Affiliations:** 1 Department of Pulmonary Medicine, School of Medicine, Koç University, Istanbul 34010, Türkiye; fuzel@kuh.ku.edu.tr (F.I.U.); fkaratas@kuh.ku.edu.tr (F.K.); 2 Department of Internal Medicine, Medicana Ataköy Hospital, Istanbul 34158, Türkiye; burak.uzel41@gmail.com; 3 Department of Molecular and Clinical Medicine, Sahlgrenska Academy, University of Gothenburg, 40530 Gothenburg, Sweden; 4 Respiratory Medicine and Allergology, Department of Clinical Sciences, Faculty of Medicine, Lund University, 22185 Lund, Sweden; 5 Division of Pulmonary, Allergy, and Critical Care Medicine, School of Medicine, University of Pittsburgh, Pittsburgh, PA 15213, USA

**Keywords:** idiopathic pulmonary fibrosis, progressive pulmonary fibrosis, antifibrotic therapy, nintedanib, pirfenidone, treatment persistence

## Abstract

**Background**: Conventional antifibrotic treatment persistence reflects medication retention but may not fully capture whether patients remain both alive and on their initial therapy. We evaluated alive and on initial treatment (AOIT) as a clinically oriented measure of treatment durability in idiopathic pulmonary fibrosis (IPF) and progressive pulmonary fibrosis (PPF). **Methods**: This retrospective single-center cohort included adults with IPF or PPF treated with pirfenidone or nintedanib between 2018 and 2026. AOIT was defined as the time from antifibrotic initiation to permanent discontinuation/switching or death while known to remain on initial treatment. Kaplan–Meier and Cox regression analyses were performed. **Results**: Among 86 patients, 37 (43.0%) experienced AOIT loss, including 26 discontinuation/switch events and 11 deaths. AOIT was 75.1%, 63.6% and 46.9% at 12, 24, and 36 months, respectively; median AOIT was 34.9 months (95% CI 21.3–48.4). AOIT did not differ between nintedanib and pirfenidone (log-rank *p* = 0.395). First-drug persistence was 80.0%, 71.7%, and 61.7% at the corresponding time points, with a median of 52.2 months (95% CI 31.2–73.2). Malignancy (adjusted HR 5.00, 95% CI 1.91–13.09, *p* = 0.001) and higher baseline DLCO (adjusted HR 1.67 per 10-percentage-point increase, 95% CI 1.21–2.29; *p* = 0.002) were associated with AOIT loss. **Conclusions**: AOIT declined over time and was numerically lower than first-drug persistence, reflecting distinct aspects of longitudinal treatment durability. AOIT may complement medication-centered persistence in real-world antifibrotic treatment assessment.

## 1. Introduction

Idiopathic pulmonary fibrosis (IPF) and progressive pulmonary fibrosis (PPF) are chronic fibrosing interstitial lung diseases characterized by progressive functional impairment and clinically important morbidity and mortality. Antifibrotic therapy has become a central component of management in IPF and, increasingly, in selected patients with PPF. Current international guidelines incorporate antifibrotic treatment within the management framework of IPF and PPF [1].

Pirfenidone and nintedanib have demonstrated efficacy in slowing functional decline in IPF, establishing antifibrotic therapy as standard pharmacological treatment for this population [2,3]. In routine clinical practice, however, long-term treatment is influenced not only by antifibrotic efficacy but also by adverse events, dose modifications, permanent discontinuation, switching between antifibrotic agents, disease progression, comorbidities, and death. Consequently, treatment persistence has emerged as an important real-world outcome reflecting the feasibility of maintaining antifibrotic therapy over time.

Recent real-world studies have highlighted substantial variability in long-term antifibrotic persistence. In the Pulmonary Fibrosis Foundation Patient Registry, persistence was evaluated according to treatment cessation, whereas the nationwide French REPEAT study defined persistence according to treatment gaps and reported a 12-month persistence rate of 70.8% among 4935 incident antifibrotic users [4,5]. Similarly, a large multicenter Polish cohort of 808 patients with IPF reported treatment discontinuation in 34.4%, without a significant difference in discontinuation rates or time to discontinuation between pirfenidone and nintedanib [6].

However, conventional treatment persistence primarily addresses a medication-centered question, namely how long the initial drug is retained. In a progressive and potentially fatal disease, this may not fully characterize the patient’s longitudinal treatment state. A patient may remain formally persistent with an initial antifibrotic until death, whereas another may discontinue or switch treatment while remaining alive. These trajectories have different clinical implications even though both influence the practical durability of initial therapy. A measure integrating survival with retention of the initially prescribed antifibrotic treatment may therefore provide information complementary to conventional medication persistence.

We therefore evaluated alive and on initial treatment (AOIT) as a clinically oriented composite state defined by continued use of the initial antifibrotic treatment while alive. The primary objective of this study was to estimate the duration and probability of remaining alive and on initial antifibrotic treatment in a real-world cohort of patients with IPF and PPF. Secondary objectives were to compare AOIT between patients initially treated with pirfenidone and nintedanib, evaluate clinical factors associated with loss of AOIT, and compare AOIT with first-drug persistence.

## 2. Materials and Methods

### 2.1. Study Design and Population

This retrospective, single-center observational cohort study was conducted at the Department of Pulmonary Medicine, Koç University. Patients discussed at the multidisciplinary interstitial lung disease meeting between 1 January 2018 and 30 August 2026, who were diagnosed with idiopathic pulmonary fibrosis (IPF) or progressive pulmonary fibrosis (PPF) and considered eligible for antifibrotic therapy were identified from the multidisciplinary meeting registry. Administrative follow-up was censored on 30 August 2026, which served as the study cutoff date for outcome ascertainment.

Diagnoses of IPF and PPF were established through multidisciplinary assessment using the diagnostic criteria applicable at the time of clinical evaluation. PPF was defined according to contemporary multidisciplinary assessment, based on evidence of progression through worsening respiratory symptoms, physiological decline, radiological progression, or a combination thereof [1].

Patients were eligible if they met all the following criteria: (1) age ≥18 years; (2) a multidisciplinary diagnosis of IPF or PPF; and (3) documented use of at least one antifibrotic agent, either pirfenidone or nintedanib. Patients were excluded if essential baseline data were unavailable or antifibrotic treatment exposure could not be verified from the available medical records.

Demographic characteristics, diagnosis, comorbidities, baseline pulmonary function, antifibrotic treatment, treatment modifications, treatment discontinuation or switching, follow-up status, and mortality data were retrospectively extracted from the medical records. Baseline pulmonary function parameters included forced vital capacity (FVC) and diffusing capacity of the lung for carbon monoxide (DLCO), expressed as percentage of predicted values.

### 2.2. Antifibrotic Treatment Exposure

Longitudinal medical records were reviewed to determine continuation of the initial antifibrotic agent, dose modifications, treatment interruptions, permanent discontinuation, and switching to the alternative antifibrotic agent. Continued initial treatment was considered ascertainable when medical records documented ongoing use of the same antifibrotic agent at follow-up visits, prescription renewals, reimbursement renewals, or multidisiplinary ILD clinic records without documentation of permanent discontinuation or switching. All death-first cases and all administratively censored cases were individually reviewed. Administrative censoring was applied only when continued use of the initial antifibrotic treatment remained ascertainable up to the administrative study cutoff date (30 August 2026) or the last documented clinical follow-up. Continued initial treatment until death or censoring was considered established when follow-up clinic records, prescription renewal records, reimbursement renewal records, or multidisciplinary ILD clinic records documented ongoing use of the same antifibrotic agent without evidence of permanent discontinuation or switching. Death was classified as a death-first AOIT event only when such documentation confirmed continuation of the initial antifibrotic treatment up to the death event. Permanent discontinuation was defined as documented cessation of the initial antifibrotic agent without subsequent resumption of the same treatment. Temporary treatment interruptions followed by resumption of the initial agent, as well as dose reductions without permanent discontinuation, were considered continued exposure and were not classified as AOIT events. When permanent discontinuation or switching occurred, the documented date of discontinuation or switching was considered the end of exposure to the initial antifibrotic agent.

### 2.3. Primary Outcome: Alive and on Initial Treatment

The primary outcome was alive and on initial treatment (AOIT), defined as the time from initiation of the first antifibrotic agent to the first occurrence of either:

1. Permanent discontinuation or switching of the initial antifibrotic agent;
2. Death while the patient was known to remain on the initial antifibrotic treatment.

The first documented component determined the AOIT event date.

Patients who permanently discontinued the initial antifibrotic agent or switched to the alternative antifibrotic before death were classified as having a first-drug discontinuation/switch event, and the date of discontinuation or switching was used as the AOIT event date.

Death was classified as a death-first AOIT event only when the patient was known to have remained on the initial antifibrotic treatment until death, without documented permanent discontinuation or switching.

Dose reduction without discontinuation or switching was not considered an AOIT event.

### 2.4. Censoring

For patients without a documented AOIT event, follow-up was censored at the last date on which continuation of the initial antifibrotic treatment could be confirmed. Continuation of treatment was not assumed during periods in which antifibrotic exposure could not be reliably ascertained.

Accordingly, if a patient was subsequently known to have died but treatment status between the last documented use of the initial antifibrotic agent and death could not be established, death was not classified as an AOIT event. Instead, the patient was censored at the last date on which continued use of the initial antifibrotic treatment could be confirmed.

Patients for whom no post-initiation confirmation of antifibrotic exposure was available were censored at the treatment-initiation date.

For patients who were subsequently documented to have died after treatment exposure became unverifiable, the exact timing of AOIT loss could not be determined because treatment status between the last confirmed treatment date and death was unknown. These patients were therefore censored at the last confirmed treatment date in the primary analysis. Alternative assumptions regarding event timing in this subgroup were explored in sensitivity analyses.

AOIT duration was calculated from the date of initiation of the first antifibrotic agent to the AOIT event or censoring date and expressed in months.

### 2.5. Components of AOIT Loss

For descriptive analyses, first AOIT events were categorized into two mutually exclusive components:

1. First-drug discontinuation/switch, defined as permanent discontinuation of the initial antifibrotic agent or switching to the alternative agent before death; and
2. Death-first, defined as death before documented discontinuation or switching while continuation of the initial antifibrotic treatment remained ascertainable.

Patients without either confirmed event were censored and were not assigned an AOIT event component.

### 2.6. Secondary Outcome: First-Drug Persistence

As a secondary analysis, first-drug persistence was evaluated separately from AOIT. For this analysis, permanent discontinuation or switching of the initial antifibrotic agent constituted the persistence event.

Death was not considered a first-drug persistence event. Patients who died while known to remain on their initial antifibrotic treatment were censored at the date of death. When treatment exposure could not be reliably established between the last documented use of the initial antifibrotic agent and a subsequent death, the patient was censored at the last date on which continuation of the initial treatment could be confirmed.

Patients who remained on their initial antifibrotic treatment without permanent discontinuation or switching were censored at the last date on which continued use of the initial treatment could be confirmed.

First-drug persistence was calculated from initiation of the first antifibrotic agent to permanent discontinuation/switching or censoring and expressed in months.

### 2.7. Sensitivity Analysis

To assess the robustness of AOIT estimates to uncertainty in treatment exposure before death, two alternative event-time scenarios were evaluated among eight patients who died after their last confirmed treatment exposure. In the first scenario, AOIT loss was assumed to occur immediately after the last confirmed exposure date, representing the earliest possible event time. In the second scenario, AOIT loss was assigned to the documented date of death, representing the latest possible event time. Under both scenarios, these patients were reclassified from censored observations to AOIT events.

Kaplan–Meier estimates of AOIT at 24 and 36 months were recalculated under each scenario. Multivariable Cox regression models were also repeated to assess the influence of these alternative assumptions on the associations of baseline malignancy and DLCO with AOIT loss.

An additional sensitivity analysis excluded the six patients for whom no post-initiation confirmation of continued antifibrotic exposure was available.

### 2.8. Statistical Analysis

Continuous variables were summarized as mean ± standard deviation or median with interquartile range, as appropriate according to their distribution. Categorical variables were summarized as frequencies and percentages.

The probability of remaining alive and on the initial treatment was estimated using the Kaplan–Meier method. AOIT probabilities were evaluated at 6, 12, 24, and 36 months, and median AOIT was reported with its 95% confidence interval (CI). AOIT curves according to the initial antifibrotic agent, nintedanib or pirfenidone, were compared using the log-rank test.

First-drug persistence was analyzed separately using the Kaplan–Meier method. Persistence probabilities at 6, 12, 24, and 36 months and median persistence with 95% CI were estimated. Persistence curves according to the initial antifibrotic agent were compared using the log-rank test. Median potential follow-up was estimated using the reverse Kaplan–Meier method, with death treated as a censored observation and the last recorded clinical follow-up treated as an event for the purpose of estimating follow-up duration.

Malignancy was defined as a baseline covariate only when the diagnosis was documented on or before the date of initiation of the first antifibrotic treatment. Malignancies diagnosed after treatment initiation were not classified as present at baseline. This definition was applied consistently in the Cox regression analyses to avoid misclassification of incident malignancies as pre-existing conditions.

Associations between baseline clinical characteristics and loss of alive and on initial treatment (AOIT) were examined using Cox proportional hazards regression with Breslow handling of tied event times. The index date was initiation of the first antifibrotic agent. Baseline DLCO was expressed as percent predicted and modeled per 10-percentage-point increase; age was modeled per 10-year increase. Malignancy was classified as a baseline covariate only when documented on or before antifibrotic initiation; malignancies first diagnosed during follow-up were not retrospectively assigned to baseline. Given the limited number of events, the final multivariable model included age, sex, baseline DLCO, initial antifibrotic agent, and baseline malignancy. Diagnosis was not retained in the final model because of the substantial imbalance between diagnosis and treatment allocation and the limited number of PPF events. Of the 86 patients included in the cohort, 5 had missing baseline DLCO values and 6 were censored at treatment initiation because no post-initiation confirmation of antifibrotic exposure was available. One patient belonged to both categories, resulting in 76 patients available for multivariable Cox regression analysis, including 33 AOIT events. Complete-case analysis was used for the multivariable model. The derivation of the final analytical population is detailed in Supplementary Table S1.

Model robustness was explored using a quadratic DLCO term, exploratory correlations of Schoenfeld residuals with event time, and alternative event-time assumptions for eight deaths occurring after the last confirmed treatment exposure. The residual correlations were exploratory and were not treated as a formal global proportional-hazards test. Results are presented as hazard ratios (HRs) with 95% confidence intervals (CIs); two-sided *p* values < 0.05 were considered statistically significant.

The proportional hazards assumption for the initial antifibrotic treatment comparison was evaluated using a treatment-by-log(time) interaction term in Cox regression. As an exploratory complementary analysis, restricted mean AOIT time was estimated at 24 and 36 months, and between-treatment differences were calculated with 95% confidence intervals obtained from 3000 stratified bootstrap resamples.

Statistical analyses were performed using IBM SPSS Statistics, version 31.0.1.0.0.

### 2.9. Ethics

The study protocol was approved by the Institutional Review Board on 17 September 2026 (approval no. 2026. 470.IRB2.273). The requirement for written informed consent was waived because of the retrospective nature of the study.

### 2.10. AI

During the preparation of this manuscript, the authors used ChatGPT 5.2 (OpenAI) to assist with language refinement, readability improvement, and figure preparation. The authors critically reviewed and edited all outputs and take full responsibility for the final content of the manuscript.

## 3. Results

### 3.1. Study Population and Treatment Course

A total of 382 patients were identified from the multidisciplinary ILD registry during the study period. Of these, 86 patients met the study eligibility criteria and received antifibrotic treatment, forming the final study cohort. Of these 86 patients, 69 (80.2%) had IPF and 17 (19.8%) had PPF. Among the 17 patients with PPF, the underlying interstitial lung diseases included 10 patients with connective tissue disease-associated ILD, 4 with chronic hypersensitivity pneumonitis, 3 with fibrotic NSIP. No lung transplantation occurred during follow-up.

Median potential follow-up, estimated using the reverse Kaplan–Meier method from antifibrotic initiation to the last recorded clinical follow-up or death, was 40.9 months (95% CI 24.6–53.5). A total of 29 deaths were documented during follow-up: 11 occurred as death-first AOIT events, 10 followed documented discontinuation or switching of the initial antifibrotic treatment, and eight occurred after the last confirmed treatment exposure, when subsequent treatment status was uncertain. Baseline demographic, clinical, pulmonary function, and antifibrotic treatment characteristics of the study population are presented in Table 1. In all, 15 (17.4%) had a documented malignancy diagnosis at the time of antifibrotic treatment initiation. An additional nine patients received a malignancy diagnosis during follow-up, resulting in 24 patients (27.9%) with malignancy recorded at any time during the observation period. Only malignancies present at treatment initiation were considered baseline malignancy in the regression analyses.

Initial antifibrotic treatment consisted of nintedanib in 44 patients (51.2%) and pirfenidone in 42 (48.8%). Treatment allocation differed substantially according to diagnosis. Among patients receiving nintedanib, 28 (63.6%) had IPF and 16 (36.4%) had PPF, compared with 41 (97.6%) and one (2.4%), respectively, among patients receiving pirfenidone (Table 1). This marked imbalance indicates limited comparability between treatment groups, particularly among patients with PPF.

### 3.2. Alive and on Initial Treatment

During follow-up, 37 of the 86 patients (43.0%) experienced loss of alive and on initial treatment (AOIT), whereas 49 patients (57.0%) were censored. Among the 37 AOIT events, 26 (70.3%) were attributed to permanent discontinuation or switching of the initial antifibrotic agent before death, and 11 (29.7%) were classified as death-first events, occurring while patients were known to remain on their initial treatment.

Overall, 29 deaths were recorded during follow-up. Of these, 11 occurred while patients were known to remain on their initial antifibrotic treatment and were classified as death-first AOIT events. Ten deaths occurred after documented permanent discontinuation or switching of the initial antifibrotic agent and therefore did not constitute first AOIT events. The remaining eight deaths occurred after the last confirmed treatment exposure, without sufficient documentation to establish whether the initial antifibrotic treatment had been continued until death. The mean interval between the last confirmed treatment record and death was 13.2 months (range, 2.4–27.8 months).

Among the 49 censored patients, 6 were censored at treatment initiation because no post-initiation confirmation of antifibrotic exposure was available. The remaining 43 patients were censored after treatment initiation. Among these, 35 underwent administrative censoring while treatment exposure remained ascertainable, whereas 8 were subsequently documented to have died after treatment exposure became unverifiable.

**Figure 1:**
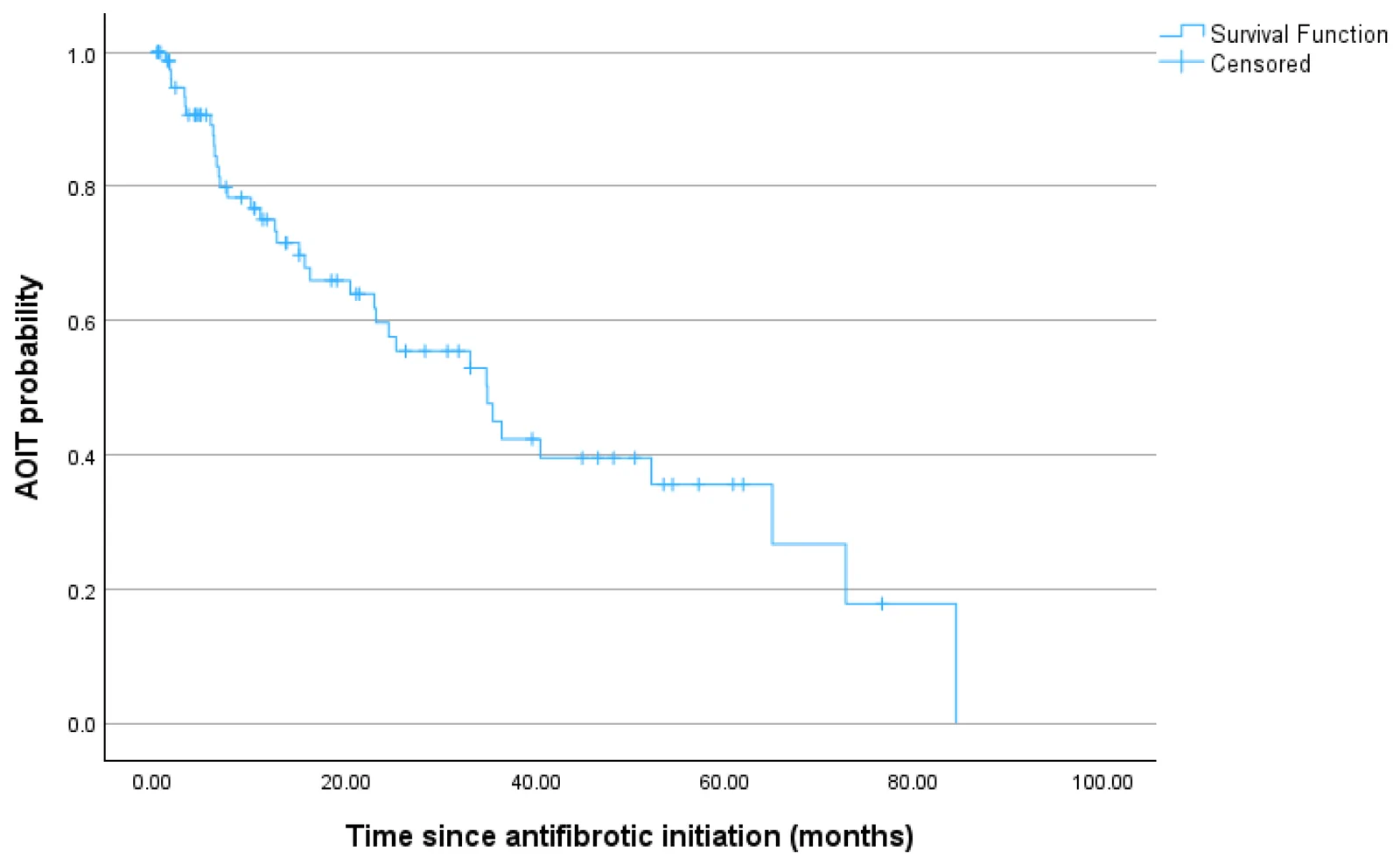
Kaplan–Meier estimate of alive and on initial treatment (AOIT) in the overall study population. Censoring marks indicate patients without an observed AOIT event.

Kaplan–Meier analysis showed a progressive decline in the probability of remaining alive and on initial antifibrotic treatment. The estimated AOIT probabilities were 86.0% at 6 months, 75.1% (95% CI 64.7–85.5) at 12 months, 63.6% (95% CI 51.4–75.8) at 24 months, and 46.9% (95% CI 32.8–61.0) at 36 months. (Figure 1).
 Number at Risk 0 Months12 Months24 Months36 MonthsOverall cohort86432817

To quantify the contribution of the individual components of AOIT loss, a competing-risk analysis was performed using the Aalen–Johansen estimator. The cumulative incidence of permanent discontinuation or switching increased from 19.3% at 12 months to 26.9% at 24 months and 34.6% at 36 months. The corresponding cumulative incidence of death-first events was 5.6%, 9.5%, and 18.5%, respectively. Thus, at 36 months, approximately one-third of the cumulative probability of AOIT loss was attributable to death-first events, highlighting the contribution of mortality to the composite endpoint (Table 2).

Among the 26 patients who experienced permanent discontinuation or switching as their first AOIT event, nine (34.6%) discontinued the initial antifibrotic agent without switching, whereas 17 (65.4%) switched to the alternative agent. Among patients initially receiving nintedanib, three discontinued without switching and nine switched; the corresponding numbers among patients initially receiving pirfenidone were six and eight, respectively.

The recorded reasons for discontinuation without switching were adverse events in seven patients and patient preference in two. Among patients who switched treatment, disease progression was recorded in eight, adverse events in seven, and the reason for switching could not be definitively established in two patients. These cases were therefore classified as having an unknown reason for treatment change.

The distribution of treatment discontinuation, switching, and their documented reasons according to the initial antifibrotic agent is presented in Table 2.

Overall, these findings indicate that loss of AOIT was driven by both treatment discontinuation or switching and death during initial treatment, with the contribution of death becoming more prominent over longer follow-up.

### 3.3. AOIT According to Initial Antifibrotic Treatment

**Figure 2:**
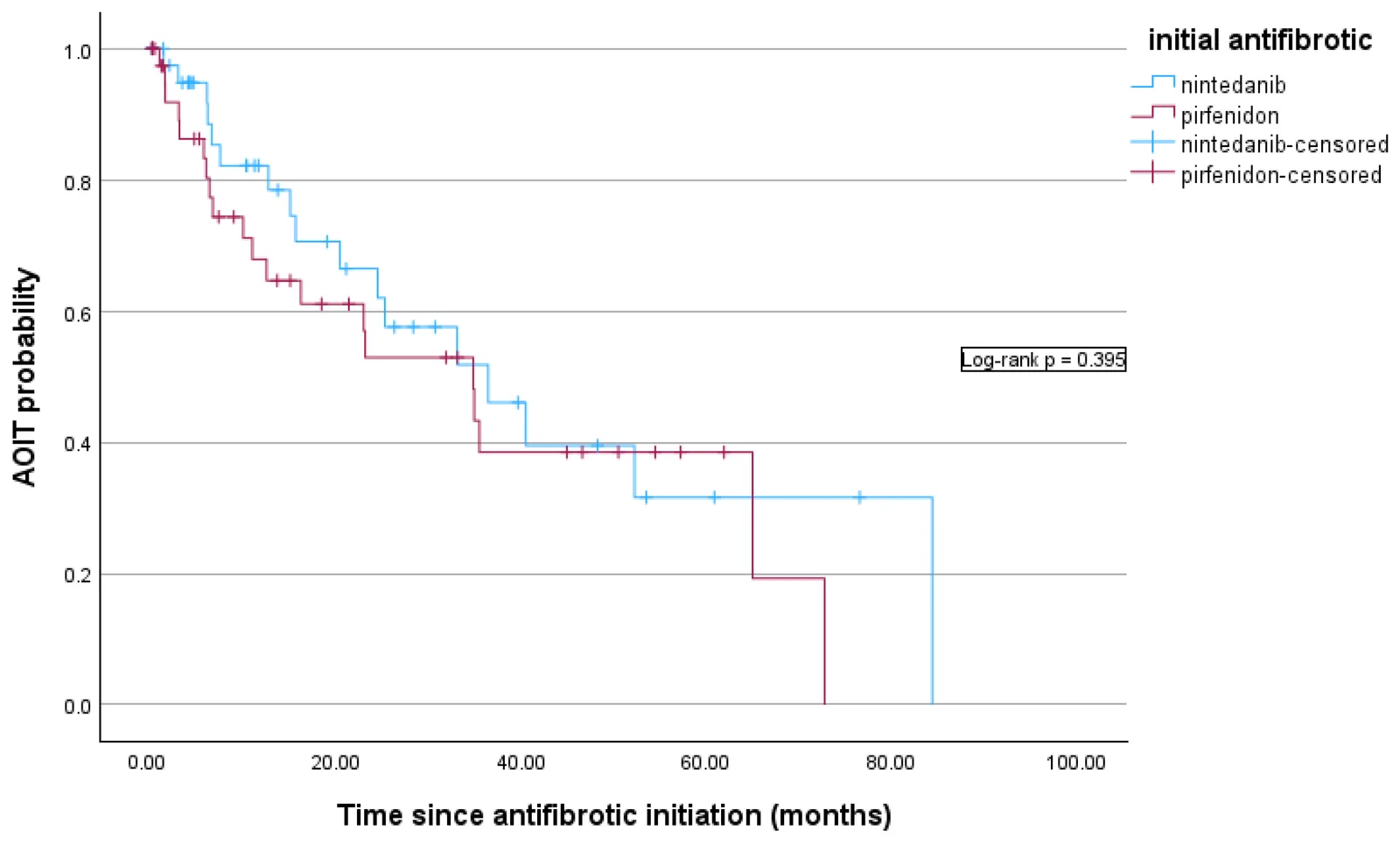
Kaplan–Meier estimates of alive and on initial treatment (AOIT) according to the initial antifibrotic agent. There was no significant difference between nintedanib and pirfenidone (log-rank *p* = 0.395).

Median AOIT was 36.4 months (95% CI, 18.8–53.9) among patients initially treated with nintedanib and 34.8 months (95% CI, 18.5–51.0) among those initially treated with pirfenidone. Kaplan–Meier curves did not differ significantly according to the initial antifibrotic agent (log-rank χ^2^ = 0.724; *p* = 0.395) (Figure 2).
 Number at Risk0 Months12 Months24 Months36 MonthsNintedanib4422159Pirfenidone4221138

The proportional hazards assumption was specifically evaluated for the initial antifibrotic treatment comparison. No statistically significant time-dependent treatment effect was detected in either the unadjusted or adjusted Cox models (treatment-by-log(time) interaction *p* = 0.672 and *p* = 0.996, respectively). In an exploratory restricted mean survival time (RMST) analysis at 24 months, restricted mean AOIT time was 19.37 months for nintedanib and 17.20 months for pirfenidone, corresponding to a between-group difference of 2.17 months (95% bootstrap CI −1.92 to 6.26). The corresponding difference at 36 months was 2.78 months (95% bootstrap CI −3.73 to 9.06). The RMST analyses were exploratory and unadjusted and should therefore be interpreted cautiously.

### 3.4. Factors Associated with Loss of AOIT

Cox analysis included 76 patients with baseline DLCO data and positive follow-up time, among whom 33 experienced AOIT loss. In the multivariable model adjusted for age, sex, initial antifibrotic agent, and malignancy present at treatment initiation, higher baseline DLCO was associated with greater hazard of AOIT loss (adjusted HR 1.67 per 10-percentage-point increase, 95% CI 1.21–2.29; *p* = 0.002). Baseline malignancy was also associated with AOIT loss (adjusted HR 5.00, 95% CI 1.91–13.09; *p* = 0.001). Age (adjusted HR 0.86 per 10 years, 95% CI 0.55–1.34; *p* = 0.494), male sex (adjusted HR 0.56, 95% CI 0.21–1.53; *p* = 0.260), and initial pirfenidone versus nintedanib (adjusted HR 1.23, 95% CI 0.58–2.63; *p* = 0.589) were not statistically significant (Table 3). Baseline characteristics of patients included in and excluded from the multivariable Cox model are compared in Supplementary Table S2.

Initial antifibrotic treatment was not significantly associated with AOIT loss in the multivariable Cox model (pirfenidone versus nintedanib: adjusted HR 1.23, 95% CI 0.58–2.63; *p* = 0.589). However, the confidence interval was wide and did not exclude clinically meaningful differences between treatments.

In a parsimonious model restricted to DLCO and baseline malignancy, the associations remained directionally consistent (DLCO: HR 1.60 per 10 percentage points, 95% CI 1.16–2.20; *p* = 0.004; malignancy: HR 4.21, 95% CI 1.76–10.06; *p* = 0.001). An exploratory quadratic DLCO term did not suggest departure from linearity on the log-hazard scale (*p* = 0.781). Exploratory Spearman correlations between Schoenfeld residuals and log event time were not statistically significant for the included covariates; however, these analyses do not exclude non-proportional hazards. Baseline DLCO values according to censoring status and first AOIT event component are presented in Supplementary Table S3. These findings require cautious interpretation because of the modest number of events and the composite nature of AOIT.

### 3.5. Sensitivity Analysis Addressing Uncertain Treatment Exposure Before Death

Eight patients who were censored at their last confirmed treatment exposure subsequently died without a documented prior permanent discontinuation or switch. To assess the potential influence of uncertainty in treatment exposure before death, these patients were reclassified as AOIT events under two alternative assumptions (Table 4 and Supplementary Table S4).

**Table 4:** Uncertain-death sensitivity analyses.

Scenario	AOIT Events	12 Months	24 Months	36 Months	Median AOIT (Months)
Primary: censor at last confirmed treatment	37	75.1%	63.6%	46.9%	34.9
Eight deaths counted at death date	45	74.4%	58.7%	40.9%	31.0
Eight deaths counted just after last confirmed use	45	72.0%	56.1%	37.8%	25.2

In the primary analysis, 37 AOIT events were observed, and the estimated probabilities of remaining alive and on initial treatment were 63.6% at 24 months and 46.9% at 36 months.

When AOIT loss was assumed to occur immediately after the last confirmed treatment exposure, the number of AOIT events increased to 45, and the corresponding AOIT probabilities decreased to 56.1% at 24 months and 37.8% at 36 months with a median AOIT of 25.2 months. When AOIT loss was assigned to the documented date of death, the corresponding estimates were 58.7% and 40.9%, respectively, with a median AOIT of 31.0 months.

Multivariable Cox regression analyses were also repeated under both alternative assumptions (Supplementary Table S5). When AOIT loss was assigned immediately after the last confirmed treatment exposure, baseline malignancy remained associated with AOIT loss (adjusted HR 2.25, 95% CI 1.16–4.37; *p* = 0.017), and the association between higher baseline DLCO and AOIT loss was preserved (adjusted HR 1.40 per 10-percentage-point increase, 95% CI 1.05–1.85; *p* = 0.020). When AOIT loss was assigned to the date of death, baseline malignancy remained associated with AOIT loss (adjusted HR 2.54, 95% CI 1.28–5.01; *p* = 0.007), and higher baseline DLCO remained associated with AOIT loss (adjusted HR 1.37 per 10-percentage-point increase, 95% CI 1.04–1.83; *p* = 0.028).

These findings indicate that absolute AOIT estimates were sensitive to assumptions regarding uncertain treatment exposure before death. However, the overall interpretation of treatment durability and the direction of the principal associations observed in multivariable regression analyses were preserved across alternative assumptions.

### 3.6. First-Drug Persistence

In a secondary analysis, first-drug persistence was evaluated using permanent discontinuation or switching of the initial antifibrotic agent as the event. Twenty-six patients (30.2%) experienced a persistence event, whereas 60 (69.8%) were censored.

Kaplan–Meier estimates of first-drug persistence were 89.9% at 6 months, 80.0% (95% CI 70.2–89.8) at 12 months, 71.7% (95% CI 59.9–83.5) at 24 months, and 61.7% (95% CI 47.2–76.2) at 36 months. Median first-drug persistence was 52.2 months (95% CI, 31.2–73.2).

Median first-drug persistence was 52.2 months (95% CI, 29.5–74.9) among patients initially treated with nintedanib and 43.4 months (95% CI, 22.9–64.0) among those initially treated with pirfenidone. First-drug persistence did not differ significantly between the two antifibrotic agents (log-rank χ^2^ = 0.329; *p* = 0.567).

First-drug persistence estimates were numerically higher than the corresponding AOIT estimates, with increasing separation over longer follow-up (Table 5).

## 4. Discussion

In this real-world cohort of patients with IPF and PPF, approximately three-quarters of patients remained alive and on their initial antifibrotic treatment at 12 months, decreasing to 63.6% at 24 months and 46.9% at 36 months. Median AOIT was 34.9 months. Among 37 AOIT events, 26 were attributable to permanent discontinuation or switching of the initial antifibrotic agent, whereas 11 represented death while the patient was known to remain on the initial treatment. AOIT did not differ significantly between patients initially treated with nintedanib and pirfenidone. In multivariable analysis, baseline malignancy and higher baseline DLCO were associated with AOIT loss, whereas age, sex and the initial antifibrotic agent were not.

A central finding of the present study was the increasing divergence between first-drug persistence and AOIT over time. Median first-drug persistence was 52.2 months, compared with 34.9 months for AOIT. These measures should not be viewed as competing estimates because they address different clinical questions: first-drug persistence reflects retention of the initial antifibrotic agent, whereas AOIT additionally incorporates whether the patient remains alive while receiving that treatment. The increasing separation over longer follow-up illustrates how medication persistence alone may incompletely describe longitudinal treatment durability in progressive fibrotic lung disease. The distinction between AOIT and conventional first-drug persistence is partly inherent to their definitions, as AOIT incorporates death as an event whereas conventional persistence focuses on treatment discontinuation or switching. Therefore, the difference between these measures should not be interpreted as independent evidence of superior prognostic performance. The competing-risk analysis further demonstrated that treatment discontinuation or switching and death contributed differently to AOIT loss over time, emphasizing the importance of interpreting the individual components alongside the composite endpoint.

This distinction is relevant in the context of contemporary real-world persistence studies. The Pulmonary Fibrosis Foundation Patient Registry characterized persistence using periods of treatment cessation, whereas the nationwide French REPEAT study used treatment gaps exceeding 60 days and reported a 12-month persistence rate of 70.8% [4,5]. These approaches appropriately quantify medication persistence but do not address the same clinical state as AOIT. Our first-drug persistence analysis therefore facilitates comparison with medication-centered outcomes, whereas AOIT provides a complementary patient-and-treatment-state perspective.

Our findings regarding the initial antifibrotic agent are consistent with previous real-world evidence. Majewski et al. reported no significant difference between pirfenidone and nintedanib in discontinuation rates or time to discontinuation [6]. Similarly, no statistically significant difference in AOIT or first-drug persistence was detected between the two agents in our cohort. In the revised multivariable Cox analysis, initial antifibrotic treatment was not significantly associated with AOIT loss (adjusted HR 1.23, 95% CI 0.58–2.63). However, this finding should not be interpreted as evidence of equivalent treatment durability. Treatment allocation was observational, and the substantial imbalance in underlying diagnosis, with 16 of 17 patients with PPF receiving nintedanib, raises concerns regarding confounding by indication and limited overlap between treatment groups. Furthermore, the wide confidence interval does not exclude clinically meaningful differences in either direction. Therefore, our findings do not establish equivalence or noninferiority between the two antifibrotic agents.

Treatment adaptation nevertheless remains an important component of real-world antifibrotic management. In a Turkish single-center cohort of 227 patients with IPF, dose reduction was required in 22.9% and switching to another antifibrotic agent occurred in 24.2% [7]. More recently, a multicenter Turkish study of 470 patients with IPF reported at least one adverse event in 70.6% and dose reduction in 18.9%, with differing adverse-event profiles between nintedanib and pirfenidone [8]. These findings reinforce that dose modification, discontinuation, and switching are common features of routine antifibrotic treatment and should not necessarily be interpreted as failure of the broader antifibrotic treatment strategy.

The inclusion of PPF alongside IPF reflects the expanding use of antifibrotic therapy across progressive fibrotic ILDs [1]. Recent real-world studies have demonstrated antifibrotic use and functional effects in both IPF and PPF populations [9,10]. Given the small PPF subgroup and wide confidence interval, our findings should not be interpreted as evidence of differential treatment durability between IPF and PPF.

Malignancy remained significantly associated with AOIT loss after adjustment, with an approximately fivefold higher hazard. However, this association should be interpreted in the context of the composite nature of AOIT. Because death constitutes one component of AOIT loss, the observed relationship may partly reflect the additional mortality burden associated with malignancy rather than reduced antifibrotic tolerability or effectiveness. Accordingly, our findings do not establish malignancy as a predictor of antifibrotic treatment failure. At antifibrotic treatment initiation, 15 patients had a documented baseline malignancy. During follow-up, an additional nine patients were diagnosed with malignancy, resulting in 24 patients with malignancy recorded at any time during observation. Most recorded malignancies were lung cancers (21 of 24 cases). Because the multivariable analysis was based on baseline malignancy status, the observed association should be interpreted in relation to malignancy present at treatment initiation rather than malignancy diagnosed during follow-up. Therefore, baseline malignancy appears to identify patients with a shorter duration of remaining both alive and on their initial antifibrotic treatment. However, because AOIT incorporates mortality as one of its components, this association may partly reflect competing mortality risk rather than a direct effect on treatment persistence. Component-specific analyses would be required to distinguish these potential mechanisms.

The association between higher baseline DLCO and increased AOIT loss was unexpected and warrants cautious interpretation. Although this association persisted in parsimonious models and alternative event-time sensitivity analyses, it should not be interpreted as evidence that better preserved gas transfer adversely affects antifibrotic treatment durability. AOIT is a composite endpoint encompassing treatment discontinuation, switching, and death, and the observed association may reflect differences in patient selection, treatment decisions, unmeasured clinical factors, or the relative contributions of individual endpoint components. Given the limited number of events and the exploratory nature of the analysis, this finding requires confirmation in independent cohorts. Several factors may contribute to this discrepancy. AOIT combines permanent discontinuation or switching with death, and the determinants of these individual components may differ. Because AOIT incorporates mortality in addition to treatment discontinuation, predictors of AOIT may differ from predictors of conventional medication persistence. Differences in patient selection, treatment indication, IPF/PPF distribution, and residual confounding may also have contributed to this finding in our relatively small cohort. The association between higher DLCO and AOIT loss should therefore be regarded as hypothesis-generating rather than evidence that better-preserved gas transfer itself confers an adverse clinical course.

An important methodological feature of the present study was the conservative handling of uncertain longitudinal treatment exposure. Continued use of the initial antifibrotic agent was not assumed during periods for which treatment status could not be reliably established. Patients without a documented AOIT event were therefore censored at the last date on which continued use of the initial therapy could be confirmed. Similarly, a subsequent death was considered an AOIT event only when the patient was known to remain on the initial antifibrotic treatment until death. Additional sensitivity analyses demonstrated that absolute AOIT estimates were sensitive to assumptions regarding uncertain treatment exposure before death. However, the overall interpretation of treatment durability and the direction of the principal associations observed in multivariable regression analyses were preserved across alternative assumptions.

### Strengths and Limitations

This study has several strengths. Treatment trajectories were reconstructed at the individual-patient level, allowing permanent discontinuation, switching, death while known to remain on initial treatment, and censoring to be distinguished according to their documented temporal sequence. The study included both IPF and PPF and had a nearly balanced distribution of initial nintedanib and pirfenidone treatment. Importantly, first-drug persistence and AOIT were evaluated within the same cohort using the same treatment history, allowing the distinction between medication retention and the broader state of remaining alive on the initial treatment to be demonstrated directly. The sensitivity analyses demonstrated preservation of the overall interpretation of treatment durability despite variation in absolute AOIT estimates under alternative assumptions regarding uncertain treatment exposure.

Several limitations should also be acknowledged. First, the retrospective, single-center design and modest sample size limit the precision and generalizability of the findings, particularly for subgroup analyses. Second, there was potential for informative censoring among patients whose treatment status could not be confirmed after their last documented exposure. Although sensitivity analyses assigning AOIT loss either to the last confirmed treatment exposure or to the subsequent date of death yielded broadly consistent findings, the absolute AOIT estimates varied across scenarios. These results indicate that incomplete treatment ascertainment may influence estimates of treatment durability, and the sensitivity analyses cannot fully eliminate this potential source of bias. Third, the PPF subgroup was relatively small, limiting robust comparisons between IPF and PPF. Fourth, the number of AOIT events limited the complexity of multivariable modeling; the adjusted analysis was therefore intentionally parsimonious and based on complete cases. Fifth, although baseline malignancy was independently associated with AOIT loss, this finding should be interpreted cautiously. AOIT is a composite endpoint incorporating treatment discontinuation or switching and death; therefore, the association does not necessarily indicate reduced antifibrotic tolerability among patients with malignancy. Furthermore, the retrospective dataset did not consistently distinguish active from previously treated malignancy or provide sufficiently detailed information on concurrent anticancer treatments. Residual confounding related to cancer activity, treatment, and prognosis cannot be excluded. Sixth, cause-specific mortality data were not consistently available; therefore, deaths could not be reliably classified as respiratory, malignancy-related, or other causes. Seventh, the retrospective dataset did not systematically capture dose reductions, temporary treatment interruptions, or detailed underlying ILD diagnoses within the PPF subgroup. Furthermore, the complete screening and exclusion log was unavailable, limiting reconstruction of the patient selection process. These limitations should be considered when interpreting treatment durability and the generalizability of the findings. Finally, AOIT is a clinically oriented composite state rather than an established or externally validated endpoint. The individual components of AOIT loss may have different determinants, and the present findings therefore require validation in larger, preferably multicenter cohorts.

## 5. Conclusions

In this retrospective real-world cohort of patients with IPF and PPF, alive and on initial treatment (AOIT) provided an exploratory composite measure integrating continued exposure to the initial antifibrotic therapy and survival. No statistically significant difference in AOIT was detected between nintedanib and pirfenidone; however, the limited sample size, imprecision of the estimates, and potential confounding by indication preclude conclusions regarding treatment equivalence. The association of baseline DLCO and malignancy with AOIT loss should be interpreted as hypothesis-generating, and comparisons between IPF and PPF remain limited by the small PPF subgroup. AOIT may complement conventional treatment persistence measures in observational research, but its reproducibility, clinical relevance, and potential utility require external validation in larger independent cohorts.

## Figures and Tables

**Table 1:** Baseline demographic and clinical characteristics according to initial antifibrotic treatment.

Characteristic	Overall Cohort (n = 86)	Nintedanib (n = 44)	Pirfenidone (n = 42)
Age at antifibrotic initiation, years	69.2 ± 7.5	68.5 ± 7.5	69.8 ± 7.5
Sex, n (%)			
Male	69 (80.2)	31 (70.5)	38 (90.5)
Female	17 (19.8)	13 (29.5)	4 (9.5)
Diagnosis, n (%)			
IPF	69 (80.2)	28 (63.6)	41 (97.6)
PPF	17 (19.8)	16 (36.4)	1 (2.4)
FVC, % predicted	82.0 ± 24.0	77.2 ± 20.2	87.0 ± 26.6
DLCO, % predicted	49.5 ± 14.5	47.2 ± 12.0	52.2 ± 16.7
Baseline malignancy, n (%)			
Present	15 (17.4)	7 (15.9)	8 (19.0)
Absent	71 (82.6)	37 (84.1)	34 (81.0)

Data are presented as mean ± standard deviation or n (%). Percentages are calculated within each treatment group. DLCO data were available for 81 patients overall (nintedanib, n = 43; pirfenidone, n = 38). Baseline malignancy was defined as malignancy documented on or before initiation of the first antifibrotic agent; malignancies first diagnosed during follow-up were excluded from this baseline classification. Abbreviations: DLCO, diffusing capacity of the lung for carbon monoxide; FVC, forced vital capacity; IPF, idiopathic pulmonary fibrosis; PPF, progressive pulmonary fibrosis.

**Table 2:** AOIT outcomes, mortality classification, competing-risk cumulative incidence, and reasons for treatment discontinuation or switching. (**A**) AOIT outcomes and mortality classification. (**B**) First AOIT discontinuation/switch events and documented reasons. (**C**) Competing-risk cumulative incidence (overall cohort).

Outcome	Overall	Nintedanib	Pirfenidone
Initial antifibrotic treatment, n	86	44	42
AOIT events, n	37	17	20
Discontinuation/switch first, n	26	12	14
Death-first AOIT events, n	11	5	6
Censored in primary AOIT analysis, n	49	27	22
Censored at treatment initiation, n	6	—	—
Censored after treatment initiation, n	43	—	—
Total recorded deaths, n	29	—	—
Death-first AOIT events, n	11	—	—
Death after documented discontinuation/switch, n	10	—	—
Death after loss of confirmed treatment exposure, n	8	—	—
Outcome/Reason	Overall	Nintedanib	Pirfenidone
Discontinuation without switching, n	9	3	6
Adverse events	7	3	4
Patient preference	2	0	2
Switch to alternative antifibrotic, n	17	9	8
Adverse events	7	4	3
Disease progression	8	4	4
Unknown reason	2	1	1
Time Since Initiation	Discontinuation/Switch First	Death-First	
12 months	19.3%	5.6%	
24 months	26.9%	9.5%	
36 months	34.6%	18.5%	

Notes. AOIT, alive and on initial treatment. The competing-risk estimates were calculated using the Aalen–Johansen estimator. Death classification is mutually exclusive: death-first, death after documented discontinuation/switch, and death after loss of confirmed exposure. Access/reimbursement reasons were not separately coded in the available dataset and could not be analyzed independently.

**Table 3:** Multivariable Cox proportional hazards analysis of factors associated with loss of alive and on initial treatment (AOIT).

Variable	Comparison/Unit	Adjusted HR (95% CI)	*p*-Value
Age at antifibrotic initiation	Per 10-year increase	0.86 (0.55–1.34)	0.494
Sex	Male vs. female	0.56 (0.21–1.53)	0.260
Baseline DLCO	Per 10-percentage-point increase	1.67 (1.21–2.29)	0.002
Initial antifibrotic treatment	Pirfenidone vs. nintedanib	1.23 (0.58–2.63)	0.589
Malignancy at treatment initiation	Present vs. absent	5.00 (1.91–13.09)	0.001

Abbreviations: AOIT, alive and on initial treatment; CI, confidence interval; DLCO, diffusing capacity of the lung for carbon monoxide; HR, hazard ratio. Note: The multivariable model included 76 patients and 33 AOIT events. DLCO was expressed as percentage of predicted values. Malignancy was defined according to status at antifibrotic treatment initiation. Hazard ratios were estimated from the revised Cox model.

**Table 5:** Comparison of first-drug persistence and AOIT.

Time Point	First-Drug Persistence	AOIT
6 months	89.9%	86.0%
12 months	80.0%	75.1%
24 months	71.7%	63.6%
36 months	61.7%	46.9%
Median, months (95% CI)	52.2 (31.2–73.2)	34.9 (21.3–48.4)
Nintedanib vs. pirfenidone, log-rank *p*	0.567	0.395

Footnote: First-drug persistence considered permanent discontinuation or switching as the event; AOIT additionally included death while the patient was known to remain on initial treatment.

## Data Availability

Data collected for the study, including de-identified individual participant data, will be made available to others within 6 months of the publication of this article for academic purposes (e.g., meta-analyses), upon request to the corresponding author (yuksel.peker@lungall.gu.se).

## Supplementary Materials

The following supporting information can be downloaded at: https://www.mdpi.com/article/10.3390/jcm15218712/s1. Table S1. Sensitivity analyses for patients who died after treatment exposure became unverifiable; Table S2. Multivariable Cox regression models under alternative assumptions regarding AOIT loss among patients who died after treatment exposure became unverifiable; Table S3: Accounting of Exclusions and Final Analytical Population for the Multivariable Cox Regression Model; Table S4. Comparison of patients included in and excluded from the multivariable Cox regression model; Table S5. Baseline DLCO according to AOIT censoring status and first-event component.

## Abbreviations

The following abbreviations are used in this manuscript:

AOIT	alive and on initial treatment
IPF	idiopathic pulmonary fibrosis
PPF	progressive pulmonary fibrosis
DLCO	diffusing capacity of the lung for carbon monoxide
FVC	forced vital capacity
CI	confidence interval
HR	Hazard ratio
SD	Standard deviation
