# Supplementary material for: Alive and on Initial Antifibrotic Treatment: Real-World Treatment Durability in Idiopathic and Progressive Pulmonary Fibrosis
Source: J Clin Med. 2026 Nov 8;15(21):8712. doi: 10.3390/jcm15218712 (PMC13763587; doi:10.3390/jcm15218712)
Supplement: Supplementary file 1 [file jcm-15-08712-s001.zip › jcm-4640463-supplementary.pdf]

Supplementary Table S1: Accounting of Exclusions and Final Analytical Population for the Multivariable Cox Regression Model

Step	n
Final study cohort	86
Missing baseline DLCO	5
Time-zero censored observations	6
Overlap between these categories	1
Excluded from multivariable Cox model	10
Included in multivariable Cox model	76
AOIT events in Cox model	33

Additional details

Characteristics	n
Missing DLCO + AOIT event	4
Missing DLCO + censored	1
Time-zero censored + missing DLCO	1

Footnote: Patients with missing baseline DLCO values and patients censored at treatment initiation because no post-initiation confirmation of antifibrotic exposure was available were excluded from multivariable Cox regression. One patient fulfilled both exclusion criteria.

Supplementary Table S2. Comparison of patients included in and excluded from the multivariable Cox regression model

Characteristic	Included in Cox model (n = 76)	Excluded (n = 10)
Age at antifibrotic initiation, years	69.2 ± 7.5	70.8 ± 5.7
Male sex, n (%)	59 (77.6)	10 (100.0)
IPF diagnosis, n (%)	60 (78.9)	9 (90.0)
Baseline malignancy, n (%)	15 (19.7)	0 (0.0)
FVC, % predicted	82.0 ± 24.0	65.7 ± 21.3

Supplementary Table S3. Baseline DLCO according to AOIT censoring status and first-event component

AOIT outcome category	n	DLCO, % predicted (mean ± SD)
Censored	48	47.7 ± 15.9
First-drug discontinuation/switch	25	52.8 ± 11.8
Death-first	8	50.5 ± 12.6
Overall	81	49.5 ± 14.5

Supplementary Table S4. Sensitivity analyses for patients who died after treatment exposure became unverifiable

Analysis	12-month AOIT	24-month AOIT	36-month AOIT	Median AOIT (months)
Primary analysis	75.1%	63.6%	46.9%	34.9
Death assigned at documented death date	74.4%	58.7%	40.9%	31.0
Death assigned immediately after last confirmed treatment exposure	72.0%	56.1%	37.8%	25.2

Sensitivity A assigned AOIT loss to the last confirmed treatment date. Sensitivity B assigned AOIT loss to the documented date of death.

Supplementary Table S5. Multivariable Cox regression models under alternative assumptions regarding AOIT loss among patients who died after treatment exposure became unverifiable

Variable	Primary analysis aHR (95% CI)	Sensitivity A aHR (95% CI)	Sensitivity B aHR (95% CI)
Age (per 10 years)	0.86 (0.55–1.34)	1.15 (0.78–1.69)	1.18 (0.80–1.74)
Male sex vs female	0.56 (0.21–1.53)	1.15 (0.43–3.05)	1.06 (0.40–2.81)
Baseline malignancy	5.00 (1.91–13.09)	2.25 (1.16–4.37)	2.54 (1.28–5.01)
Baseline DLCO (per 10-percentage-point increase)	1.67 (1.21–2.29)	1.40 (1.05–1.85)	1.37 (1.04–1.83)
Initial antifibrotic treatment (Pirfenidone vs Nintedanib)	1.23 (0.58–2.63)	1.43 (0.72–2.84)	1.25 (0.63–2.52)

Sensitivity A assigned AOIT loss to the last confirmed treatment date. Sensitivity B assigned AOIT loss to the documented date of death. All sensitivity models used the same covariate set as the primary multivariable model (age, sex, baseline malignancy, baseline DLCO, and initial antifibrotic treatment).

Analysis	Patients	AOIT events
Primary	76	33
Sensitivity A	81	41
Sensitivity B	75	41
